# Distinct Roles of Monocyte Subsets in Cancer

**DOI:** 10.3390/cells14241982

**Published:** 2025-12-13

**Authors:** Maria Amparo Sahagun Cortez, Wolf Eilenberg, Christoph Neumayer, Christine Brostjan

**Affiliations:** Division of Vascular Surgery, Department of General Surgery, Medical University of Vienna, University Hospital Vienna, 1090 Vienna, Austria; maria.sahaguncortez@meduniwien.ac.at (M.A.S.C.); wolf.eilenberg@meduniwien.ac.at (W.E.); christoph.neumayer@meduniwien.ac.at (C.N.)

**Keywords:** cancer, tumor microenvironment, monocyte subsets

## Abstract

While the distinct roles of lymphocyte populations are well characterized in adaptive immunity, the phenotypic and functional diversity of innate immune cells is less explored. In recent years, subsets of monocytes have gained attention, as prominent shifts in population frequencies have been observed in disease states such as cancer. This narrative review summarizes current knowledge of the distribution and functional differences among the three major monocyte subsets (classical, intermediate, non-classical) in tumor settings. It includes rare populations, such as neutrophil-like, CD56+, and Tie2-expressing monocytes. Scientific evidence indicates that the phenotypical and functional heterogeneity of monocyte subsets determines their roles in either preventing cancer development or supporting the progression of disease through a remarkable diversity of mechanisms. Of note, alterations in the distribution of monocyte subsets and their functional reprogramming have been identified as drivers of cancer progression. While changes in monocyte frequencies have limited diagnostic biomarker potential for cancer detection, they may reflect the progression of disease and response to therapy. Based on subset-specific properties, distinct monocyte populations are increasingly recognized as promising targets of cancer immunotherapy. Yet novel strategies targeting monocyte populations must consider the risk of treatment reversal given the high plasticity of these cells.

## 1. Introduction

The innate immune response is responsible for the so-called “non-specific” removal of pathogens, the coordination of the inflammatory response, and the restoration of tissue homeostasis [1]. However, failing to reinstate equilibrium may contribute to cancer development and progression, supported by the sustained release of pro-inflammatory cytokines [2]. The resulting oxidative stress can give rise to DNA damage, thereby activating tumor-promoting genes or down-regulating tumor-suppressor genes, and the genomic alterations lead to an increasingly malignant state [3,4]. Monocytes are innate immune cells of the myeloid lineage, and their functions include tissue homeostasis, host defense, and regulation of inflammation [5]. These cells are produced in the bone marrow by hematopoietic stem and progenitor cells (HSPCs) during homeostasis; their production is enhanced during emergency monopoiesis, which is the response to infectious and inflammatory stimuli, and in tumor development [5]. The heterogeneity of monocytes is based on distinct gene expression patterns, as reflected by the level of CD14 and CD16 in humans and Ly6C and CD43 in mice. Accordingly, three major subtypes have been defined in humans: CD14++CD16- as classical or inflammatory monocytes, CD14++CD16+ as intermediate monocytes, and CD14+CD16++ as non-classical or patrolling monocytes [6]. Monocyte subsets arise from the conversion of classical monocytes into non-classical monocytes in the blood circulation, with intermediate monocytes representing a transition state with yet distinct properties [7]. Transcriptomic analyses comparing mouse and human monocytes linked Ly6C^Hi^CD43^Low^ to the classical CD14++CD16- monocytes, and Ly6C^Low^CD43^Hi^ monocytes to the non-classical monocytes, despite some differences in gene expression and surface markers [8,9].

Apart from the three major types of monocytes, additional subsets, mostly of lower frequency, have been characterized. For example, a monocyte with neutrophil characteristics has been identified during monopoiesis and classified as a neutrophil-like monocyte [10]. Moreover, the expression of the neural cell adhesion molecule, also known as NCAM or CD56, or the angiopoietin receptor Tie2 has been detected in distinct monocyte populations [11,12]. These subsets are referred to as CD56+ monocytes (CD14+CD56+) and Tie2-expressing monocytes (TEMs), respectively.

Thus, the present review aims to summarize the role and functions of the above-mentioned monocyte subsets in primary stages of cancer development as well as tumor progression and metastasis. It addresses the mechanisms involved in the recruitment of distinct subsets to the tumor microenvironment and the phenotypical and functional changes the monocytes undergo due to interactions with cancer cells. Finally, the biomarker potential of monocytes in cancer and novel immunotherapeutic strategies targeting monocytes are summarized.

## 2. Materials and Methods

The following narrative review searched the PubMed database for literature (without date-of-publication limitations) using keywords and terminology that represent the main topics discussed in each section. Articles were screened by title and then abstract to select those relevant to the specific functions of each subset at different stages of tumorigenesis. These included mechanisms of recruitment into the tumor microenvironment, the phenotypical and functional reprogramming of monocytes induced by cancer cells, the role of monocytes in tumor growth and metastasis, along with their potential as diagnostic biomarkers or their role in cancer therapy. Aiming to focus on monocyte populations in solid cancer, publications on myeloid leukemia were excluded from the review.

## 3. Monocyte Biology and Subsets

### 3.1. Monopoiesis

Monocytes are innate immune cells of the myeloid lineage that arise from hematopoietic stem cells in the bone marrow, as predominantly characterized in the murine system and confirmed for the human setting [5,13]. While myeloid cells are constantly supplied to tissues for immune surveillance in the steady state, a reserve is maintained in the bone marrow that can be rapidly mobilized in response to pathogens and inflammatory cytokines during emergency myelopoiesis [14].

The classical model of adult hematopoiesis describes the development of granulocytes, monocytes, and dendritic cells (DCs) from common myeloid progenitors (CMPs) that give rise to granulocyte-monocyte progenitors (GMPs) and monocyte-DC progenitors (MDPs) [15,16]. Thus, monocytes were described to descend from the committed monocyte progenitor (cMoP) derived from MDPs, resulting in a CMP-GMP-MDP-cMoP-monocyte hierarchical model of monopoiesis [13].

**Figure 1:**
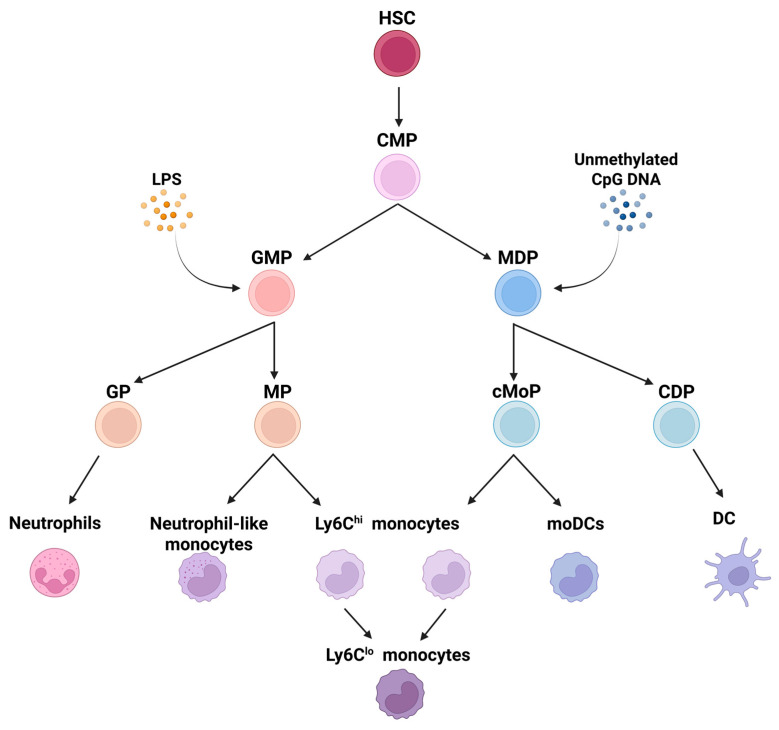
Hierarchical model of monopoiesis where GMPs and MDPs give rise to monocytes. While both progenitors yield classical (Ly6C^Hi^) monocytes that may further differentiate into non-classical (Ly6C^Low^) monocytes, GMPs also produce neutrophils through granulocyte progenitors (GPs), and MDPs give rise to dendritic cells via the common dendritic cell progenitor (CDP). Of note, monocyte-derived dendritic cells (moDCs) arise exclusively from cMoPs, while neutrophil-like monocytes are MP-derived. Regarding pathogen triggers of monopoiesis, lipopolysaccharide (LPS) was found to primarily stimulate neutrophil and monocyte production from GMPs, whereas unmethylated CpG DNA induces the production of monocytes and conventional DCs derived from MDPs. (Illustration is based on [10,17,18,19,20] and was created in BioRender at the following site: https://BioRender.com). Abbreviations: CDP, common dendritic cell progenitor; cMoP, committed monocyte progenitor; CMP, common myeloid progenitor; DC, dendritic cell; GMP, granulocyte-monocyte progenitor; GP, granulocyte progenitor; HSC, hematopoietic stem cell; LPS, lipopolysaccharide; MDP, monocyte-DC progenitor; MP, monocyte progenitor; moDCs, monocyte-derived dendritic cells.).

However, Yáñez et al. proposed a different model in 2017 [10], suggesting that progenitors make a lineage decision earlier in the hierarchy and monocytes may indeed derive from two distinct progenitors, GMPs and MDPs, where GMPs give rise to a monocyte progenitor (MP) distinct from the described cMoP. Importantly, classical monocytes developing from MPs and cMoPs differ in their gene expression patterns, functions, and tissue homing properties [17]. The newly proposed model also suggests that MDPs do not originate from GMPs but are directly derived from CMPs (Figure 1) [10,17,18]. Furthermore, GMPs have the capacity to produce monocytes, neutrophils, and a distinct population of Ly6C^Hi^ monocytes with neutrophil-like characteristics, while MDPs give rise to monocytes, DCs, and monocyte-derived dendritic cells (moDCs), lacking neutrophil potential [10,19,20]. In a disease setting, the production of classical monocytes from the GMPs or MDPs also depends on the type of stimulus. For instance, lipopolysaccharide (LPS) primarily induces neutrophil and monocyte production derived from GMPs, whereas unmethylated CpG DNA triggers the release of monocytes and conventional DCs derived from MDPs (Figure 1) [10]. Yet to date, the selective mobilization of monocytes via the GMP or MDP lineage is largely unexplored for conditions with chronic inflammation, such as cancer.

The selective activation of lineage-determining transcription factors at different stages of hematopoiesis regulates the development and production of monocytes [21,22]. The classical (Ly6C^Hi^) monocyte fate from MP or cMoP progenitor populations is dependent on the sequential expression of PU.1, IRF8, and KLF4 transcription factors [16], whereas the further differentiation from classical into non-classical (Ly6C^Low^) monocytes is regulated by the transcription factor Nur77 [10,23].

Interestingly, Rhee et al. [24] recently identified four distinct monocyte populations whose fate was defined by chromatin accessibility profiles at or before the GMP or MDP level, showing minimal plasticity of monocyte descendants once differentiation had begun. From the four monocyte populations identified, only two presented similar characteristics to the established classical and non-classical subsets, thus suggesting further analysis of the other two populations to determine their relationship to the monocyte subsets (classical, intermediate, and non-classical) in the current classification scheme [10,23,24,25].

### 3.2. Monocyte Heterogeneity

Monocytes are primarily defined as mononuclear phagocytes that constitute the first line of innate immune response. However, the variety of functions exerted by these cells includes tissue homeostasis, initiation and propagation of host responses to pathogens, regulation of inflammation, and resolution of immune responses before excessive tissue damage takes place [6,26,27]. Functional heterogeneity among monocytes with distinct predominant features may thus seem plausible. The heterogeneity of monocytes was initially discovered to be reflected by the level of expression of LPS co-receptor (CD14) and FcγRIII receptor (CD16); accordingly, as mentioned above, circulating monocytes have been classified into three different subtypes: CD14++CD16− classical, CD14++CD16+ intermediate, and CD14+CD16++ non-classical monocytes [6,28]. Importantly, the heterogeneity of the monocyte populations leads them to perform distinct functions in physiological and pathological settings, as in the regulation of tumor growth and metastasis [29,30].

Classical monocytes account for about 85% of peripheral blood monocytes and present the highest phagocytosis potential, as well as a more pro-inflammatory phenotype compared to the other subsets [28]. They are characterized by high gene expression of CD14, C-C chemokine receptor (CCR) 2, and pro-inflammatory mediators, and may also support functions in wound healing, thus making them highly versatile to mediate immune functions and repair after tissue injury [28]. Classical monocytes are promptly recruited to infection and injury sites, where they have the functional plasticity to differentiate into monocyte-derived macrophages and DCs, as well as stimulate the innate and adaptive immune response [31,32]. Due to these characteristics, classical monocytes are the main source of tumor-associated macrophages (TAMs), as they constitute the majority of monocytes recruited to the primary tumor and metastatic sites from the bone marrow in response to the C-C chemokine ligand (CCL) type 2 interaction with its receptor CCR2 [32,33]. Classical monocytes have a half-life in blood circulation of less than 24 h in humans and mice, compared to 7 days in humans and close to 48 h in mice of the non-classical subset [7,31,34]. Moreover, classical human monocytes have the potential to develop into intermediate monocytes within 24 h after being in circulation before fully transitioning into non-classical monocytes 96 h later [31]. The percentage of differentiated intermediate and non-classical monocytes rises in disease conditions such as cancer [35].

Thus, the remaining monocytes found in circulation are further divided into two populations: about 5% intermediate (CD14++CD16+) and 10% non-classical (CD14+CD16++) monocytes [28,36]. The intermediate subset is characterized by its antigen-presenting and inflammatory functions, as well as a high proangiogenic potential among monocyte subsets. Although intermediate monocytes present similar levels of C-X3-C motif chemokine receptor 1 (CX3CR1) as the non-classical population, they do not actively patrol the vasculature [28,36,37]. Intermediate monocytes present a high gene expression of MHC class II, the class II invariant chain (CD74), and HLA-DO isotype involved in MHC class II antigen processing; therefore, this subtype plays a primary role in the stimulation and activation of T cells [28].

Based on their gene expression profile, non-classical monocytes are involved in complement and Fc gamma-mediated phagocytosis and adhesion, as well as patrolling the vascular endothelium through the activation of CX3CR1 in the steady state [28,37]. This subset reacts strongly to viral signals and is also associated with the wound-healing process [37,38,39]. Of note, non-classical monocytes are considered the primary monocyte population involved in the anti-cancer response, secreting tumor necrosis factor α (TNF-α) and IL-12 upon interaction with tumor cells [26,40].

A number of additional monocyte populations of low frequency have been characterized in the context of cancer, which are mostly subsets of the three major classes. For example, the so-called Tie2-expressing monocytes (TEMs) can be detected in peripheral blood, with the highest representation in the intermediate monocyte subset [12,41]. TEMs are also found in solid tumors and represent a major population after TAMs [12,42]. The expression of the Tie2 receptor in monocytes confers them pro-angiogenic properties through the binding of angiopoietins to the receptor [43]. This subpopulation of myeloid cells promotes neovascularization in tumor tissue by the expression of mediators such as vascular endothelial growth factor A (VEGF-A) or transforming growth factor beta (TGF-β) [44,45].

Another tumor-associated monocyte subset is characterized by the surface expression of CD56: Although the neural cell adhesion molecule CD56 is mostly associated with natural killer (NK) cells, where it is expressed at high levels, this phenotypic marker has been identified on other immune cells, like CD4+ and CD8+ T cells, DCs, and monocytes [46]. CD56+ monocytes (CD14+CD56+) are capable of infiltrating the tumor, lysing tumor cells after activation with interferon (IFN) α, and stimulating cytotoxic functions of DCs [11].

Of note, a monocyte subpopulation expressing the CD66b marker, which is typically associated with granulocytes, has been termed neutrophil-like monocytes (HLA-DR+CD14+CD66b+) [47]. Findings indicate a dual role of this subset by presenting both pro- and anti-inflammatory properties, thus either resulting in anti-tumoral functions or support of tumor development [47,48]. Of note, this monocyte subset shows high capacity for phagocytosis, cell migration, and matrix adhesion [47].

## 4. The Effect of Monocyte Subsets in Cancer

### 4.1. Monocyte Recruitment to Tumor Sites

**Figure 2:**
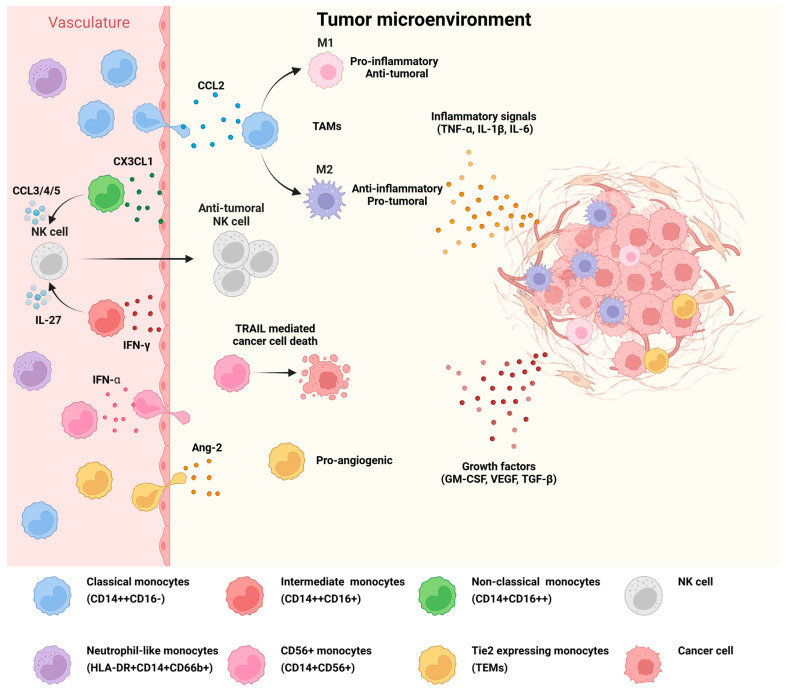
Recruitment mechanisms for monocyte subset infiltration into the tumor microenvironment. Classical monocytes infiltrate the tumor site in response to a chemokine gradient, primarily driven by the CCL2-CCR2 axis. After infiltration, they differentiate into pro-inflammatory M1 or anti-inflammatory M2 TAMs depending on the TME-derived signals. Non-classical monocytes are recruited to the inflammatory site mainly via CX3CL1-CX3CR1 signaling, leading to a feedback loop of CCL3/4/5 release and the recruitment and activation of anti-tumoral NK cells. Intermediate monocytes can be recruited via CCR2 or CX3CR1 and can similarly trigger NK cell responses when activated by IFN-γ. CD56+ monocytes infiltrate the TME upon activation by IFN-α and promote cancer cell death. In contrast, TEM recruitment is mediated by angiopoietin 2 (Ang-2) release from the hypoxic TME and Ang-2 binding to the Tie2 receptor on TEMs. (The illustration summarizes literature findings [11,12,28,36,44,50,51,52,54,60,63,64] and has been created in BioRender at the following site: https://BioRender.com). Abbreviations: Ang-2, angiopoietin 2; CCL2/3/4/5, C-C chemokine ligand 2/3/4/5; CCR2, C-C chemokine receptor 2; CX3CL1, C-X3-C motif chemokine ligand 1; CX3CR1, C-X3-C motif chemokine receptor 1; GM-CSF, granulocyte-monocyte colony stimulating factor; HLA, human leukocyte antigen; IFN, interferon; IL, interleukin; NK, natural killer; TAMs, tumor associated macrophages; TEMs, Tie-2 expressing monocytes; TGF-β, transforming growth factor beta; TNF-α, tumor necrosis factor alpha; TRAIL, tumor necrosis factor-related apoptosis-inducing ligand; VEGF, vascular endothelial growth factor).

Tumors are considered chronic inflammatory areas and sites of tissue remodeling, thus activating the innate immune response by the constant release of inflammatory signals (TNF-α, interleukins such as IL-1β, IL-6) and growth factors (granulocyte-monocyte colony-stimulating factor, GM-CSF, VEGF-A, TGF-β) that shape the tumor microenvironment (TME) and support tumor progression and metastasis [49]. Circulating monocytes migrate to the TME in response to a chemokine gradient. For instance, CD62L/CD62L ligands, CCR2/CCL2, VEGF receptor R1/VEGF-A, and CX3CR1/CX3CL1 receptor-ligand pairs have been most prominently associated with the recruitment of monocytes to the tumor microenvironment [50]. Given that the various monocyte subsets express distinct receptors, studies indicate differences in their recruitment to tumor sites and in their reprogramming (Figure 2) [50]. Particularly, CD62L, CCR2, and VEGFR1 are highly expressed in classical monocytes but not in non-classical monocytes [51,52], while the expression of CX3CR1 is characteristic of non-classical monocytes [28,36].

Thus, the recruitment of classical monocytes from peripheral blood to the tumor site is predominantly mediated by the CCL2-CCR2 chemotactic axis, where monocytes further differentiate into tumor-associated macrophages (TAMs) once they reach the TME [51,53]. The plasticity of classical monocytes allows their phenotypic differentiation into M1 or M2 macrophages [54]. The development into M1 macrophages leads to increased production of pro-inflammatory cytokines (IL1-β, TNF-α, and IL-6), and support of the adaptive anti-tumor activity [55]. M1 macrophages can phagocytose dead cancer cells, upregulate antigen-presentation leading to activation of CD8+ T cell anti-tumoral responses, and induce tumor cell death by producing nitric oxide. Thus, high M1 macrophage infiltration is correlated with a positive outcome in cancer [56]. However, the anti-tumoral response of M1 macrophages mainly takes place during the early stages of tumor development, while the tumor microenvironment progressively reprograms them into an M2 phenotype [52,57]. M2 macrophages, also known as anti-inflammatory macrophages, suppress immune functions in the TME, induce angiogenesis, and promote tumor growth and metastasis [54,58].

Conversely, non-classical monocytes patrol the vasculature where they clear cancer cell material. The presence of pro-inflammatory cytokines, such as TNF-α, IL-1, and IFN-γ, upregulates the expression of CX3CL1 on the endothelium [59], leading to the recruitment of non-classical monocytes to the tumor site. Activation of non-classical monocytes through CX3CR1 leads to the upregulation of CCL3, CCL4, and CCL5 chemokines, resulting in the recruitment and activation of anti-tumoral NK cells, which contribute to cancer cell death, while this monocyte subset is not able to directly kill tumor cells [60,61]. Of note, this mechanism has primarily been characterized for the recruitment of non-classical monocytes to the lung. Moreover, non-classical monocytes were found to be of importance in the activation and generation of effector memory CD8+ T cell responses during early metastatic seeding in lungs [62].

Since the intermediate monocyte subset represents a transition state between the classical and non-classical monocyte subsets, it shares properties of both populations and expresses CCR2, CCR5, and CX3CR1 (Figure 2) [36,63]. Thus, in one respect, IFN-γ-induced intermediate monocytes were found to inhibit lung cancer metastasis by promoting NK cell expansion through IL-27 [64]. In contrast, the recruitment of the intermediate subset by the complement component C5a-regulated CCL2/CCR5 axis enhanced their infiltration into the pleural cavity and supported the development of malignant pleural effusion [65].

Regarding the low-frequency monocyte subsets mentioned above, IFN-α is known to activate CD56+ monocytes to infiltrate into the tumor and lyse cancer cells through the tumor necrosis factor-related apoptosis-inducing ligand (TRAIL) pathway and via T cell activation [11]. TEM recruitment into the tumor is promoted by angiopoietin 2 (Ang-2), which is upregulated by the TME due to the occurrence of hypoxic regions, inducing the process of angiogenesis (Figure 2) [12,44]. Although the chemokine receptors and the recruitment mechanism of neutrophil-like monocytes to tumor tissue are not completely understood, the growth factors secreted by cancer cells seem to shift monopoiesis towards the GMP pathway, thereby increasing the release of this subset [48,66]. Neutrophil-like monocytes are significantly elevated in the blood of patients with various cancers (breast, colorectal, lung, pancreatic, and gastric). In contrast, they are hardly detectable in the circulation of healthy individuals [47].

### 4.2. Reprogramming of Monocyte Subsets to Promote Tumorigenesis

While monocyte differentiation towards an immunosuppressive phenotype occurs once they infiltrate the TME, the reprogramming of monocytes by the tumor may already be initiated in circulation. Cancer cells can affect circulating monocytes either directly or indirectly (through intermediary cells of the TME), releasing cytokines and extracellular vesicles into the bloodstream [67]. It was recently shown that systemic inflammation triggered by the tumor results in the preconditioning of peripheral monocytes at the transcriptional and epigenetic level, which blocks pro-inflammatory and anti-tumoral responses [67]. This may, at least in part, explain the origin and regulation of so-called monocytic myeloid-derived suppressor cells (Mo-MDSCs), which are well known to be elevated in the blood of cancer patients [68]. The transcriptional reprogramming of monocytes due to tumor signals (such as IL-6, IL-10, and TGF-β) mainly relies on activation of the signal transducer and activator of transcription 3 (STAT3), leading to the upregulation of genes associated with anti-inflammatory cytokines, pro-angiogenic molecules, and growth factors [67,69]. Mo-MDSCs are characterized by their capacity to release nitric oxide and reactive nitrogen species, the upregulation of PD-L1, reduced secretion of inflammatory cytokines, and elevated expression of arginase-1, which inhibits T cell anti-tumoral responses [70]. Moreover, Mo-MDSCs promote tumor progression and facilitate metastasis by supporting tumor angiogenesis through the release of VEGF-α [27,66,70]. In line with this, lower Mo-MDSC counts in circulation correlate with a better survival prognosis in cancer patients [70,71].

The chemotactic signals that guide monocytes to the tumor may also drive their reprogramming. For instance, tumor-derived CCL2 promotes M2 polarization. In addition, the uptake of tumor microparticles by macrophages leads to the enhanced secretion of CCL2 to recruit further monocytes that predominantly differentiate into pro-tumor macrophages [53,72]. A high concentration of CCR2+ monocytes, which is a characteristic of the classical monocyte subset, within the TME is correlated with T cell suppression and promotion of tumor growth in several cancer models. This interaction highlights how immunosuppressive mechanisms already initiated during monocyte recruitment contribute to tumor progression (Table 1) [51].

The process is further amplified, as briefly mentioned in the previous chapter, when the TME polarizes infiltrating classical monocytes to differentiate into M2 macrophages by, e.g., release of IL-4 and IL-10, anti-inflammatory cytokines. This leads to the expression of CD163 and CD206, which are involved in maintaining homeostasis and secreting anti-inflammatory factors that further suppress the immune response [73]. M2 macrophages also express high levels of VEGF, which promotes the delivery of oxygen and nutrients to the tumor by supporting the process of neovascularization. In addition, the M-CSF signaling pathway maintains the immunosuppressive state of the TME by mediating the constant recruitment and survival of TAMs [74]. In contrast to M1 macrophages, M2 macrophages have a poor antigen-presenting function and suppress innate and adaptive anti-tumor responses; thus, high M2 infiltration is associated with poor cancer patient outcomes [57,75]. Similarly, TEMs infiltrate the tumor and support the establishment of a functional vasculature [44]. The up-regulation of Ang-2 and VEGF in the TME enhances the pro-angiogenic function of this monocyte subset, leading to the formation of tumor vasculature and tissue remodeling, which were shown to promote tumor growth and metastasis in several cancer types [43,76,77].

**Table 1:** Pro- and anti-tumor functions of reprogrammed monocyte subsets.

Monocyte Subset	Phenotype	Function	Ref.
Classical (CD14++CD16−)	Anti-tumoral (M1 phenotype)	Production of inflammatory cytokines Induction of cancer cell death Activation of anti-tumoral CD8+ T cells	[50,55,56]
	Pro-tumoral (Mo-MDSCs or M2 phenotype)	Production of anti-inflammatory cytokines Support of tumor angiogenesis Inhibition of T cell anti-tumoral response	[27,57,67,68,69,70]
Non-classical (CD14+CD16++)	Anti-tumoral	Patrolling of the vasculature and engulfment of tumor material Recruitment and activation of NK cells Activation of CD8+ T cells	[59,60,61,62]
Intermediate (CD14++CD16+)	Anti-tumoral	IFN-γ-activated monocytes induce NK cell expansion	[64]
	Pro-tumoral	C5a-activated monocytes support tumor proliferation, cell survival, migration, and EMT	[65]
TEMs (Tie2-expressing monocytes)	Pro-tumoral	Drivers of tumor angiogenesis and tissue remodeling by the release of growth factors	[43,44,76,77]
Neutrophil-like monocytes (HLA-A2+ CD14+CD66b+)	Pro-tumoral	Induction of NK cell dysfunction via IL-10 secretion	[48,78]
	Anti-tumoral	Co-stimulation of CD4+ and CD8+ T cell activation	[47]
CD56+ monocytes (CD14+CD56+)	Anti-tumoral	Tumor cell lysis via the TRAIL signal T cell activation	[11]

Abbreviations: EMT, epithelial-to-mesenchymal transition; HLA, human leukocyte antigen; IFN-γ, interferon gamma; IL-10, interleukin 10; Mo-MDSCs, monocytic myeloid-derived suppressor cells; NK, natural killer; Ref, references; TEMs, Tie-2 expressing monocytes; TRAIL, tumor necrosis factor-related apoptosis-inducing ligand.

Despite the many ways in which tumor-driven preconditioning and reprogramming of (predominantly classical) monocytes to a pro-tumoral phenotype, the recruited monocytes can also differentiate into an immunostimulatory phenotype and promote anti-tumor immunity by T or NK cells [79]. Especially interferon-responsive monocytes, such as intermediate monocytes and CD56+ monocytes, can induce cancer cell death either directly or indirectly by activating anti-tumoral NK cells, thereby restricting tumor spread [11,64]. However, when intermediate monocytes are activated via C5a, they secrete IL-1β and facilitate tumor cell proliferation, migration, and epithelial-to-mesenchymal transition (EMT), while reducing tumor cell apoptosis [65].

A similar “Janus-faced profile” has emerged for the subset of CD66b+ neutrophil-like monocytes. While anti-tumoral effects by supporting CD8+ and CD4+ T cell activation have been observed [47], this subset was found to interfere with NK cell anti-tumor responses by secreting IL-10 and inducing the expression of the immune checkpoint molecule TIGIT (T cell immunoreceptor with immunoglobulin and ITIM domain) in NK cells of colorectal cancer patients [48]. However, the variation in the reported functions of neutrophil-like monocytes might be due to differences in immunophenotyping strategies used by the authors, as well as differences between cancer types [78]. Further studies are certainly necessary to clarify the role(s) of neutrophil-like monocytes in cancer.

### 4.3. Monocyte Contribution to Cancer Progression and Metastasis

The various subsets of monocytes are also involved in the process of tumor metastasis, where cancer cells lose their cell–cell adhesions, acquire migratory properties, and invade the extracellular matrix to enter the bloodstream and establish a metastatic site.

Thus, the generation of immunosuppressive cells derived from classical monocytes, namely Mo-MDSC and M2 macrophages, not only supports primary tumor growth but further facilitates the cancer cell colonization of the metastatic site [72,80,81]. One of the mechanisms involved includes the secretion of matrix metalloproteinase-9 (MMP-9) by M2 macrophages, which promotes the establishment of the pre-metastatic niche, as well as the extravasation of cancer cells to the metastatic site since it induces EMT of cancer cells and supports the invasion phenotype of cancer cells [81,82,83]. Additionally, Mo-MDSCs were described to facilitate liver colonization and immune escape of cancer cells, in association with TME recruitment and activation of regulatory T cells [84]. Notably, immunosuppressive monocytes are more frequently found in patients with metastatic disease [70], and metastasis is a marker of poor prognosis across all cancer types [58,85].

Tissue remodeling and, in particular, neovascularization are prerequisites for the intra- and extravasation of (circulating) tumor cells to spread via the vascular or lymphatic routes. These processes are not only supported by MMP-9, but also by MMP-2 and Ang-2 [81,86]. Hence, TEMs have been proposed to play a central role in this context [12,44]. Since they exhibit potent proangiogenic and immunosuppressive activity mediated by the Tie-2 and VEGFR signaling pathways, this monocyte subset supports metastatic colonization by facilitating the extravasation of cancer cells and decreasing T cell proliferation [87].

In contrast to these pro-metastatic effects, non-classical monocytes were reported to inhibit tumor metastasis to the lung by the above-mentioned mechanism of activating and recruiting NK cells to kill cancer cells and scavenge tumor microparticles [60]. Similarly, IFN-γ-induced intermediate monocytes decrease lung metastasis by initiating anti-tumoral NK cell expansion through IL-27 [64].

## 5. Monocytes as Cancer Biomarkers and Their Subset-Specific Responses to Therapy

### 5.1. Circulating Monocytes as Biomarkers for Disease Development, Stage, and Prognosis

When screening the literature, the development of cancer is generally associated with a shift in the common monocyte subsets, mostly resulting in a higher proportion of the CD16+ populations (intermediate and/or non-classical monocytes) and a decrease in classical monocytes. Yet, alterations in monocyte subset distribution vary across different types and stages of cancer and have been evaluated for their diagnostic and prognostic biomarker potential (Table 2).

For instance, the level of CD16+ monocyte subsets is increased in the peripheral blood of breast cancer patients compared with healthy individuals [88,89,90,91]. While the combined fraction of non-classical and intermediate monocytes has been negatively correlated to breast cancer stage and tumor size [89], patients with high levels of intermediate monocytes tended to present more circulating tumor cells, thus correlating with poor prognosis in metastatic disease [90]. However, breast cancer subtype as well as the post- or premenopausal status of the patient are additional variables that may shift the distribution of monocyte subsets [92]. Furthermore, monitoring Mo-MDSC levels in breast cancer patients could be used to assess the disease progression from early breast cancer to the metastatic stage, since higher levels of Mo-MDSC were associated with disseminated breast cancer [93].

In comparison, ovarian cancer was found to influence the distribution of circulating monocyte subsets, where the intermediate monocyte population was amplified, and the classical monocyte population was decreased compared to healthy controls; however, the surface marker expression levels of the monocyte subsets remained unchanged [94]. The expansion of intermediate monocytes in the blood of ovarian cancer patients correlated with the inhibition of cytotoxic CD8+ T cells and poor prognosis [94]. Similarly, patients with oral squamous cell carcinoma showed a comparable shift, with elevated intermediate and reduced classical monocytes [95].

Colorectal cancer patients presented a high concentration of intermediate monocytes, in contrast to the classical and non-classical subsets, when compared to healthy individuals. As this shift was mainly observed in patients with localized as opposed to metastatic disease, it could indicate that intermediate monocytes are induced at the early stages of cancer development [35].

The frequency of TEMs was also investigated for various cancer types and reported to be increased in cervical cancer [42], renal cell carcinoma [76], colorectal cancer [12], hepatocellular carcinoma [96], and breast cancer [77] patients compared with healthy controls, thus suggesting circulating TEMs as a biomarker for these cancers. Of note, TEM levels frequently correlated with cancer progression and poor prognosis, in line with TEMs promoting tumor angiogenesis leading to neoplastic growth and metastasis [42,43,96,97].

Regarding the CD56- or CD66b-expressing monocyte subsets, cancer patients presented with a higher blood cell count of CD56+ monocytes compared to healthy controls, specifically at the early stages of tumor development, which could indicate that the tumor might downregulate CD56+ monocytes during progression [11]. Also, the level of CD66b+ monocytes was found to be particularly upregulated in cancer patients compared to healthy controls [47,67]. However, considering that a shift in monocyte subsets is not unique to the state of cancer but is quite commonly observed for other types of diseases with acute or chronic inflammation, the diagnostic marker potential of monocyte subsets seems restricted, while longitudinal monitoring of patients for prognostic or predictive purposes may show more promise.

**Table 2:** Monocyte subsets as blood biomarkers for diagnosis or prognosis of different cancer types.

Cancer Type	Monocyte Subset	Diagnostic and Prognostic Information	Ref.
Breast cancer	Non-classical monocyte increase	Inverse correlation between non-classical monocyte count and tumor size	[89]
	Intermediate monocyte increase	Correlation with a high amount of circulating tumor cells and poor prognosis	[90]
Ovarian cancer	Intermediate monocyte increase	Association with CD8+ T cell inhibition and poor prognosis	[94]
Colorectal cancer	Intermediate monocyte increase	Observed at early stages of cancer development as opposed to tumor metastasis	[35]
Several cancer types	TEM increase	Correlation with cancer progression and poor prognosis	[12,42,76,77,96]
	Neutrophil-like monocyte increase	Correlation with tumor development; uncommon in healthy individuals	[47,67]
	CD56+ monocyte increase	Observed at early stages of cancer development	[11]
	Mo-MDSC elevation	Correlation with cancer progression and poor prognosis	[70,71]

Abbreviations: Mo-MDSC, monocytic myeloid-derived suppressor cell; Ref, references; TEM, Tie-2 expressing monocyte.

### 5.2. Monocyte Effects in Tumor Therapy

Treatment options to reduce tumor burden, including the primary tumor and the metastatic site, comprise surgery, chemotherapy, radiation therapy, targeted therapy, and immunotherapies [98,99,100,101,102,103]. Although the treatment strategy varies according to the type and stage of cancer, surgery is the primary curative approach and is often preceded or followed by other types of therapy to enhance effectiveness [98]. These types of (mostly systemic) treatments exert differential effects on monocyte populations, which may support or counteract treatment efficacy.

Chemotherapy, for instance, was reported to enhance CCL2-driven (classical) monocyte recruitment and differentiation into tumor-promoting M2 macrophages, thereby reducing the adaptive anti-tumor immunity [104,105]. Another major drawback of chemotherapy is the recruitment of TEMs by the tumor to promote neovascularization, thus contributing to metastatic relapse [106]. As observed with chemotherapy, radiotherapy similarly alters the phenotype of CCL2/CCR2-recruited monocytes towards tumor-supporting Mo-MDSCs, which promotes radioresistance [107]. However, in a recent publication on conformal (as opposed to non-conformal) radiotherapy of breast cancer, the positive treatment outcome was mediated by monocyte recruitment and activation in an IFN type I-dependent manner, leading to CD8+ T cell anti-tumor responses [108].

Regarding the most commonly used immunotherapies of anti-PD-1 and anti-PD-L1 immune checkpoint inhibitors, cancer patients with high counts of classical monocytes in peripheral blood showed less favorable responses to treatment and had decreased progression-free survival [109].

A particularly surprising observation was made in the context of anti-angiogenic cancer therapy: Even though non-classical monocytes are known to trigger anti-tumoral activity by NK cell recruitment [60], the subset was skewed toward an immunosuppressive phenotype following anti-VEGFR2 cancer therapy in a mouse model of colorectal cancer [110,111]. Essentially, CX3CL1 was upregulated by anti-angiogenic therapy, resulting in the accumulation of non-classical monocytes, which recruited immunosuppressive neutrophils and blocked adaptive immune responses via IL-10 secretion [110,111]. Thus, co-administration of anti-angiogenic therapy with immunomodulatory strategies to block the influx and reprogramming of non-classical monocytes was suggested to potentially improve treatment outcome.

## 6. Conclusions and Future Perspective

Monocyte subsets play distinct roles in the development and treatment of tumors and their metastatic sites. Therefore, novel therapeutic strategies might build on this knowledge and target specific monocyte populations and their TME-reprogramming to interfere with tumor progression. Since immunosuppressive, tumor-promoting monocytes are mainly derived from the classical subset, it has been suggested that targeting the CSF1/CSF1R [112,113] or CCL2/CCR2 [52] axis by antibodies might have therapeutic benefit. For example, in a hepatocellular carcinoma model, blockade of the CCL2/CCR2 axis inhibited the recruitment of classical monocytes, thereby blunting M2-polarization of TAMs and the TAM-mediated suppression of CD8+ T cells [114]. However, in a metastatic breast cancer model with CCL2 inhibition, an overshooting counterreaction was observed when treatment was interrupted. Animals demonstrated increased rates of metastasis and death, linked to the release of monocytes from bone marrow, enhanced mobilization of tumor cells from the primary site, and elevated angiogenesis driven by IL-6 and VEGF [115]. This cautions to carefully consider the therapeutic targets and tools when attempting to reprogram circulating monocyte populations for the benefit of cancer patients, since the high plasticity of monocytes may also carry inherent risks of treatment reversal.

Alternatively, adoptive transfer of purified monocyte subsets could more effectively target tumor cells. While adoptive transfer of monocyte-derived and tumor peptide-laden dendritic cells has been initiated decades ago [116], clinical attempts to directly transfer monocytes are fairly recent and have shown promising first results [117]: The activation of total monocytes with interferon was applied to successfully elicit anti-cancer responses of monocytes. These therapeutic effects might be even more pronounced when selecting distinct monocyte subsets with known anti-tumor capacity (such as the non-classical, CD56+ or neutrophil-like monocytes) for adoptive transfer. However, lessons learned from dendritic cell therapy [118] caution against compensatory tumor reactions such as upregulation of checkpoint protein expression. Hence, future therapy approaches are likely to involve a combination of monocyte therapy with other treatments, possibly with checkpoint inhibitors.

## Data Availability

Not applicable.

## Abbreviations

Ang-2	Angiopoietin 2
CCL2	C-C Chemokine Ligand 2
CCR2	C-C Chemokine Receptor 2
CDP	Common Dendritic Cell Progenitor
cMoP	Committed Monocyte Progenitor
CMP	Common Myeloid Progenitor
CX3CR1	C-X3-C Motif Chemokine Receptor 1
DC	Dendritic Cell
GM-CSF	Granulocyte–Monocyte Colony-Stimulating Factor
GMP	Granulocyte–Monocyte Progenitor
GP	Granulocyte Progenitor
HSPC	Hematopoietic Stem and Progenitor Cell
IFN	Interferon
IL	Interleukin
LPS	Lipopolysaccharide
MDP	Monocyte–Dendritic Cell Progenitor
MMP	Matrix Metalloproteinase
moDC	Monocyte-Derived Dendritic Cell
Mo-MDSC	Monocytic Myeloid-Derived Suppressor Cell
MP	Monocyte Progenitor
NK	Natural Killer
TAM	Tumor-Associated Macrophage
TEM	Tie2 Expressing Monocyte
TGF-β	Transforming Growth Factor Beta
TIGIT	T cell Immunoreceptor with Immunoglobulin and ITIM Domain
TME	Tumor Microenvironment
TNF-α	Tumor Necrosis Factor Alpha
TRAIL	Tumor Necrosis Factor-Related Apoptosis-Inducing Ligand
VEGF	Vascular Endothelial Growth Factor
